# Supplementary material for: Major distribution shifts are projected for key rangeland grasses under a high-emission scenario in East Africa at the end of the 21st century
Source: Commun Earth Environ. 2024 Oct 17;5(1):600. doi: 10.1038/s43247-024-01731-x (PMC11486655; doi:10.1038/s43247-024-01731-x)
Supplement: Supplementary file 2 — Supplementary information [file 43247_2024_1731_MOESM2_ESM.pdf]

Supplementary information to "Major distribution shifts are projected for key rangeland grasses under a high-emission scenario in East Africa at the end of the 21st century"

Martina Messmer[1,2,9*], Sandra Eckert[3,4], Amor Torre-Marin Rando[5], Mark Snethlage[5], Santos J. González-Rojí[1,2,10], Kaspar Hurni[3], Urs Beyerle[6], Andreas Hemp[7], Staline Kibet[8], and Thomas F. Stocker[1,2]

[1] Climate and Environmental Physics, University of Bern, Sidlerstrasse 5, Bern, 3012, Bern, Switzerland.

[2] Oeschger Centre for Climate Change Research, University of Bern, Sidlerstrasse 5, Bern, 3012, Bern, Switzerland.

[3] Centre for Development and Environment, University of Bern, Mittelstrasse 43, Bern, 3012, Bern, Switzerland.

[4] Department of Geography, University of Bern, Hallerstrasse 12, Bern, 3012, Bern, Switzerland.

[5] Institute of Plant Sciences, University of Bern, Altenbergrain 21, Bern, 3012, Bern, Switzerland.

[6] Institute for Atmospheric and Climate Science, ETH Zurich, Universitätstrasse 16, Zurich, 8092, Zurich, Switzerland.

[7] Department of Plant Systematics, University of Bayreuth, Universitätsstrasse 30, 95440 Bayreuth, Germany.

[8] Land Resource Management and Agricultural Technology Department, College of Agriculture and Veterinary Sciences, University of Nairobi, P.O Box 29053-00625, Nairobi, Kenya

[9] Present address: Geoscience and Remote Sensing, Faculty of Civil Engineering and Geosciences, Delft University of Technology, Stevinweg 1, 2628 CN, Delft, The Netherlands

[10] Present address: Department of Physics, University of the Basque Country, Barrio Sarriena s/n, 48940, Leioa, Spain

*Corresponding author: m.messmer@tudelft.nl

Table S1: Grass species selected for the modelling, the number of presence and absence points, and their relevance as fodder plants.

Grass species	Presence points	Absence points	Fodder relevance
Cenchrus ciliaris	286	316	Palatable to livestock<br>Its dominance reduces the abundance of unpalatable species<br>Provides good value forage<br>Most preferred species in dry season pastures due to its abundance[1]
Cynodon dactylon	538	285	Valuable pasture and fodder grass<br>Preferred dry season grazing forage, as it improves the livestock production (highly nutritive forage)[1,2]
Cynodon plectostachyus	85	125	Palatable to livestock<br>Valuable as fodder<br>Good nutrition value
Digitaria macroblephara	132	224	Palatable to livestock<br>High digestibility, ability to increase milk and weight of livestock<br>Can withstand continuous grazing[1,3]
Digitaria milanjiana	147	312	Highly palatable to livestock
Cenchrus mezianus (Pennisetum mezianum)	103	321	Relatively unpalatable when mature, but consumed by livestock during fodder shortage (important to bridge dry periods)<br>Most preferred species in dry season pastures due to its abundance[1]
Pennisetum stramineum	21	349	Relatively unpalatable when mature, but consumed by livestock during fodder shortage (important to bridge dry periods)
Themeda triandra	313	269	Contributes substantially to pasture resilience<br>Decline is associated with deterioration of grazing value<br>Highly palatable to livestock[1]

Table S2: Variable importance in descending order for the BRT model. The models' TSS are indicated below the names of the grass species. The most important predictor is indicated as the relative importance in per cent. The last column shows the average over all species, therefore no TSS is available. All variable abbreviations can be found in Table S4.

C. ciliaris		*C. dactylon*		*C. plectostachyus*		*D. macroblephara*		*D. milanjiana*		*C. mezianus*		*T. triandra*		Overall	
TSS	0.53	TSS	0.57	TSS	0.52	TSS	0.49	TSS	0.51	TSS	0.51	TSS	0.52	TSS	
TC	37.10	Bio2	37.30	Bio14	35.10	Bio2	36.30	TC	20.80	TC	28.70	TC	19.50	TC	23
Bio14	13.00	Bio12	13.60	TC	22.30	TC	25.90	Bio1	18.50	Bio2	18.20	Bio4	17.40	Bio2	18
Bio1	11.50	Bio4	12.50	Bio1	10.60	Bio4	9.70	Bio4	12.80	Bio14	12.60	Bio1	11.70	Bio14	11.5
Bio2	9.10	HFI	7.10	CEC	9.30	Bio1	9.00	Bio2	11.60	Bio4	10.50	Bio2	10.70	Bio4	10.6
slope	6.70	TC	6.90	slope	5.70	slope	6.60	Bio12	8.80	slope	9.50	Bio12	10.50	Bio1	10.2
Bio4	6.20	Bio1	5.70	Bio4	5.40	Bio12	4.90	Bio14	6.10	d2ww	5.10	Bio14	7.10	Bio12	7.2
d2ww	4.70	Bio14	5.40	Bio12	4.20	CEC	2.50	CEC	5.90	Bio1	4.70	CEC	6.90	slope	6.1
Bio12	4.60	slope	4.40	Bio2	3.10	d2ww	1.90	HFI	5.30	Bio12	3.50	d2ww	5.70	CEC	5
CEC	3.10	CEC	4.30	d2ww	2.80	HFI	1.60	slope	5.20	HFI	3.20	slope	4.90	d2ww	3.9
HFI	3.00	d2ww	2.30	HFI	1.40	Bio14	1.20	d2ww	4.80	CEC	2.80	HFI	4.60	HFI	3.7
Texture	1.10	Texture	0.40	Texture	0.10	Texture	0.40	Texture	0.30	Texture	1.30	Texture	1.20	Texture	0.7

Table S3: Area of different grassland types in 100 square kilometres, separated into absent, no change, contraction, expansion, and overall change, obtained from the BRT model. The last column indicates the relative change in per cent of the relevant species' area of presence under present climate conditions.

Species	Absent [100 km^2]	No change [100 km^2]	Contraction [100 km^2]	Expansion [100 km^2]	Change [100 km^2]	Change [%]
C. ciliaris	877	6417	379	686	307	5.0
C. dactylon	1271	5116	1327	644	-683	-11.8
C. plectostachyus	1555	5310	1067	427	-640	-10.8
D. macroblephara	1009	6500	409	441	32	0.5
D. milanjiana	1502	5455	302	1100	798	17.1
C. mezianus	3273	2706	1946	434	-1512	-35.8
T. triandra	1823	4304	1061	1172	112	2.7

Table S4: List of all bioclimatic variables and important covariates considered for the species distribution modelling. Highlighted in bold are the predictors selected for the final model.

**Bioclimatic variables**	**Other important covariates**
**Bio 1: annual mean temperature**	elevation (ALOS World 3D) (elev)[4]
**Bio 2: annual mean diurnal temperature range**	**slope (ALOS World 3D)**[4]
Bio 3: isothermality	**distance to waterways (d2ww)**
**Bio 4: temperature seasonality**	distance to waterbodies (d2wb)
Bio 5: maximum temperature of warmest month	**human footprint index (HFI)**[5]
Bio 6: minimum temperature of coldest month	**Hansen's global tree cover 2010 (TC)**[6]
Bio 7: temperature annual range	AfriSoils exchange aluminium (exAl)[7]
Bio 8: mean temperature of wettest quarter	**SoilGrids texture**[8]
Bio 9: mean temperature of driest quarter	SoilGrids pH[8]
Bio 10: mean temperature of warmest quarter	SoilGrids salinity[8]
Bio 11: mean temperature of coldest quarter	SoilGrids soil depth[8]
**Bio 12: annual precipitation**	**SoilGrids cation exchange capacity (CEC)**[8]
Bio 13: precipitation of wettest month	SoilGrids soil class[8]
**Bio 14: precipitation of driest month**	SoilGrids clay percentage (clay)[8]
Bio 15: precipitation seasonality	SoilGrids silt percentage (silt)[8]
Bio 16: precipitation of wettest quarter	SoilGrids soil organic carbon (SOC)[8]
Bio 17: precipitation of driest quarter	SoilGrids nitrogen (N)[8]
Bio 18: precipitation of warmest quarter	Landsat normalized difference vegetation index (NDVI)[9,10]
Bio 19: precipitation of coldest quarter	

Table S5: Overview of the regional climate simulations. The bold simulations build the basis for the species distribution modelling.

Period	WRF simulation	Input data
1981–2010	present	CESM
2071–2100	future	CESM
1999–2018	**ERA5**	ERA5
2071–2100	**corrected future climate simulation**	ERA5 + (future − present)

Table S6: List of the used CMIP5 models, listing their acronyms, the full model name and the horizontal resolution of the atmospheric component given in °lat × °lon.

Acronyms	Full model name	°lat × °lon
ACCESS1-0	Australian Community Climate and Earth System Simulator	1.9 × 1.2
BCC-CSM1-1	Beijing Climate Center Climate System Model	2.8 × 2.8
CESM1-CAM5-FV2	Community Earth System Model	1.2 × 0.9
CNRM-CM5	Centre National de Recherches Météorologiques	1.4 × 1.4
GFDL-ESM2G	Geophysical Fluid Dynamics Laboratory Earth System Model	2.5 × 2.0
GISS-E2-R	Goddard Institute for Space Studies ModelE2	2.5 × 2.0
HadGEM2-AO	Hadley Centre Global Environment Model version 2 atmosphere-ocean configuration	1.9 × 1.2
HadGEM2-CC	Hadley Centre Global Environment Model version 2 carbon cycle configuration	1.9 × 1.2
HadGEM2-ES	Hadley Centre Global Environment Model version 2 Earth system configuration	1.9 × 1.2
INMCM4	Institute of Numerical Mathematics Climate Model version 4	2.0 × 1.5
IPSL-CMSA-LR	Institut Pierre Simon Laplace Climate Model low resolution	3.7 × 1.9
MIROC-ESM-CHEM	Model for Interdisciplinary Research on Climate Earth System Model with chemistry component	2.8 × 2.8
MIROC-ESM	Model for Interdisciplinary Research on Climate Earth System Model	2.8 × 2.8
MIROC5	Model for Interdisciplinary Research on Climate atmosphere-ocean	1.4 × 1.4
MPI-ESM-LR	Max Planck Institut Earth System Model low resolution	1.9 × 1.9
MRI-CGCM3	Meteorological Research Institute Coupled Global Climate Model version 3	1.1 × 1.1
NorESMI-M	Norwegian Earth System Model medium resolution	2.5 × 1.9

Table S7: List of each of the three tuned parameters of the RF (left) and BRT (right) model, for each of the seven grass species.

Species	RF model			BRT model		
	mtry	ntree	nodesize	tree.complexity	learning.rate	bag.fraction
C. ciliaris	2	1600	1	20	0.01	0.75
C. dactylon	1	200	1	30	0.005	0.75
C. plectostachyus	3	100	2	5	0.01	0.5
D. macroblephara	2	500	1	10	0.005	0.75
D. milanjiana	1	500	3	20	0.01	0.5
C. mezianus	2	100	4	20	0.005	0.5
T. triandra	1	200	5	15	0.01	0.75
C. ciliaris	2	100	1	30	0.01	0.75

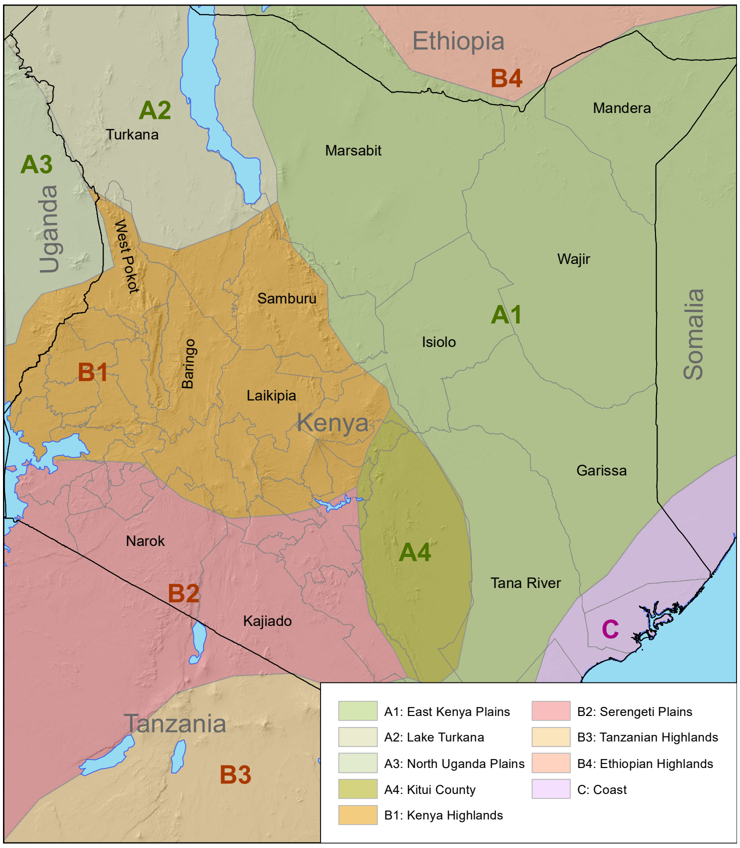

Figure S1: Definition of our physiographic units of East Africa. The lowlands of the north and east are shaded in greenish colours (A1–A4), the highlands are shaded in reddish colours (B1–B4), and the coast is shaded in purple (C). For more details compare Section “Study region”.

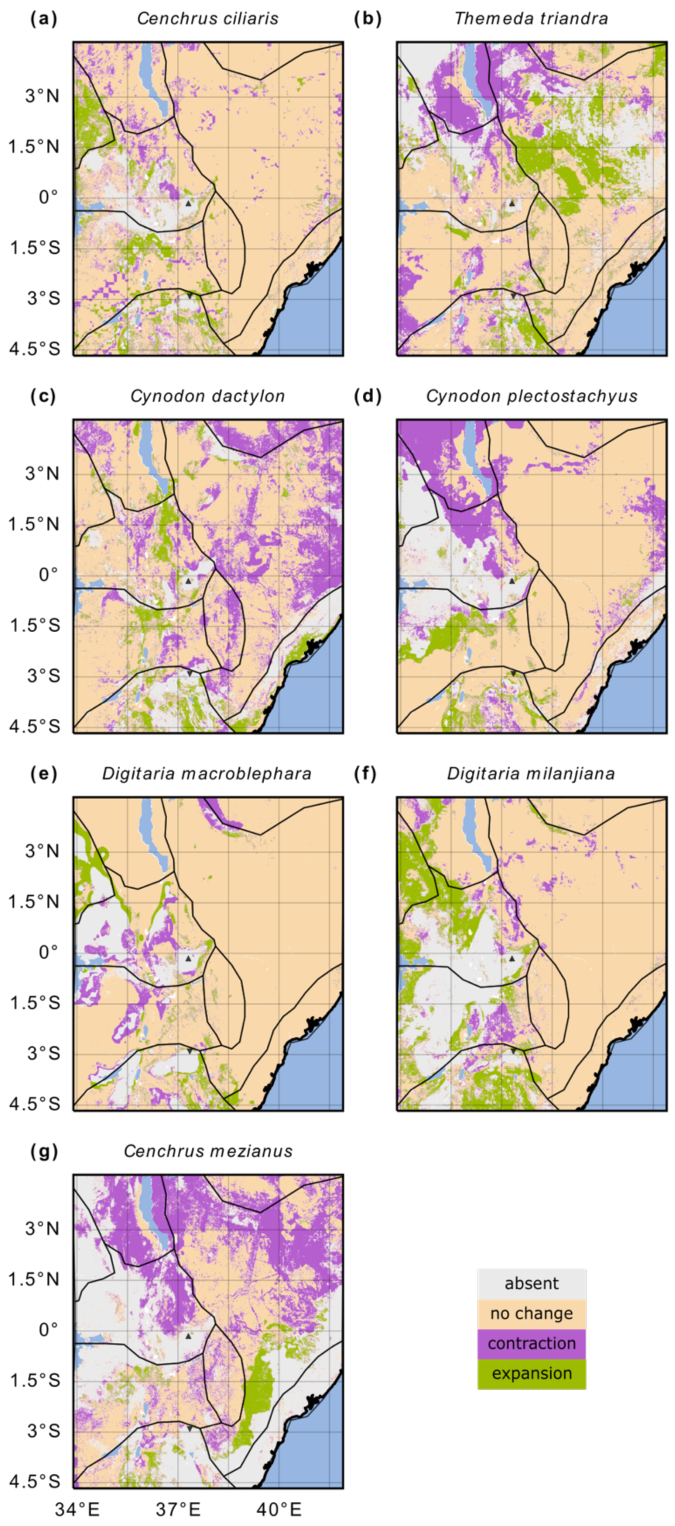

Figure S2: Changes in the occurrence of the seven grass species between the present and the future predicted by the BRT model. (a) *C. ciliaris*, (b) *T. triandra*, (c) *C. dactylon*, (d) *C. plectostachyus*, (e) *D. macroblephara*, (f) *D. milanjiana*, and (g) *C. mezianus*. The upward-pointing black triangle indicates the location of Mount Kenya, and the downward-pointing one marks Mount Kilimanjaro. The grey, beige, pink and green shaded areas indicate absence, no change, range contraction and range contraction, respectively, for each species under future climate conditions. For more details see Section "Species data and species distribution models".

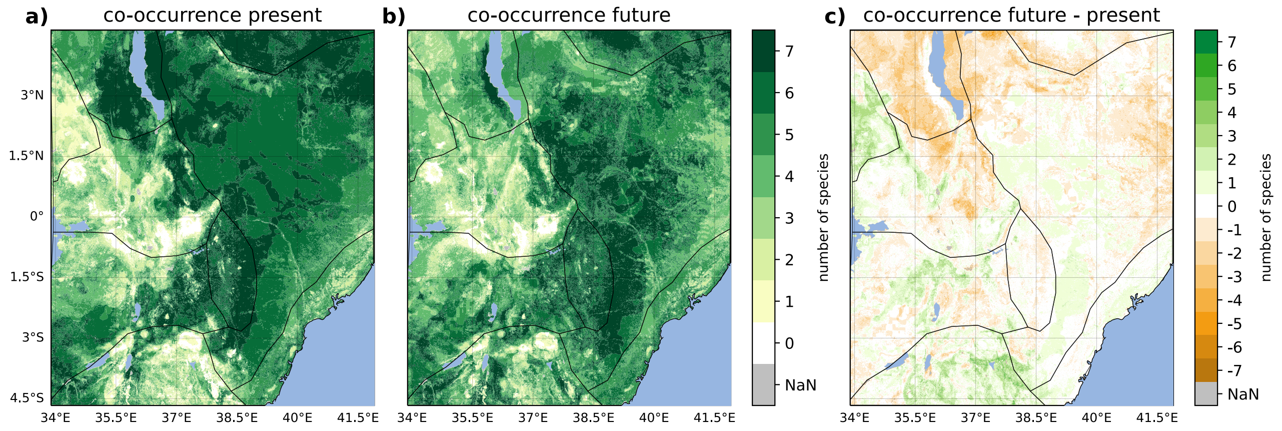

Figure S3: Predicted co-occurrence of the seven grass species under (a) present and (b) future climate conditions. c) indicates the change in the co-occurrence of the grass species under future climate compared to present climate conditions obtained from the BRT model. Orange (green) shadings in panel c) indicate a decline (increase) in the number of species populating the same area.

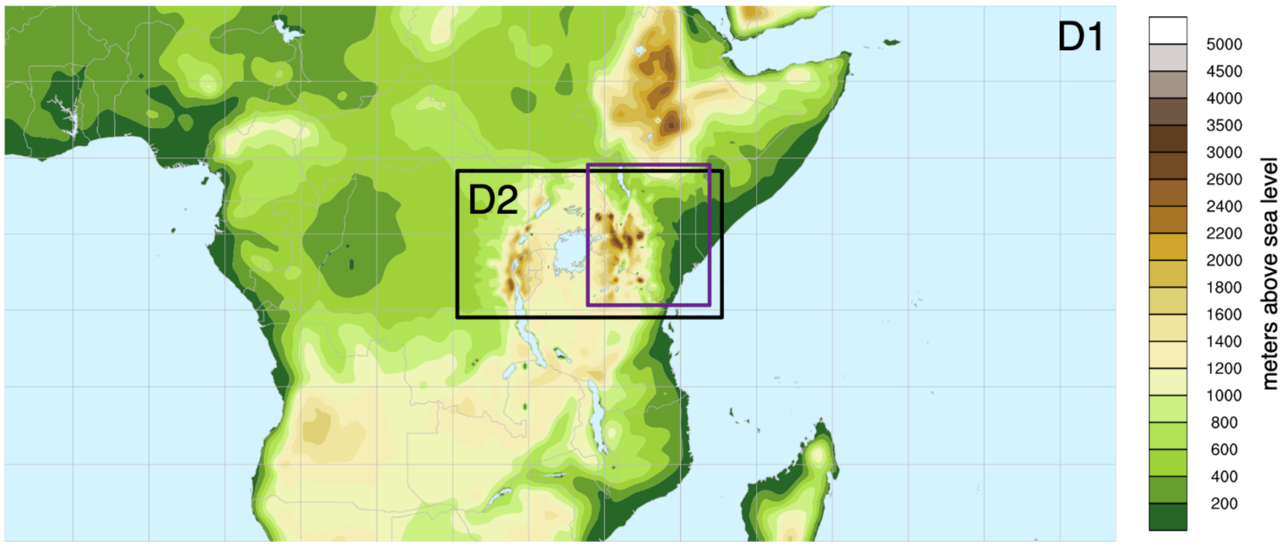

Figure S4: Domain 1 (D1, 27 km spatial resolution) and domain 2 (D2, 9 km, black box) used for the different WRF simulations. The shading indicates elevation in metres above sea level using the WRF topography Global Multi-resolution Terrain Elevation Data (GMTED2010) provided by USGS. The purple box denotes the study area for the grass species assessment.

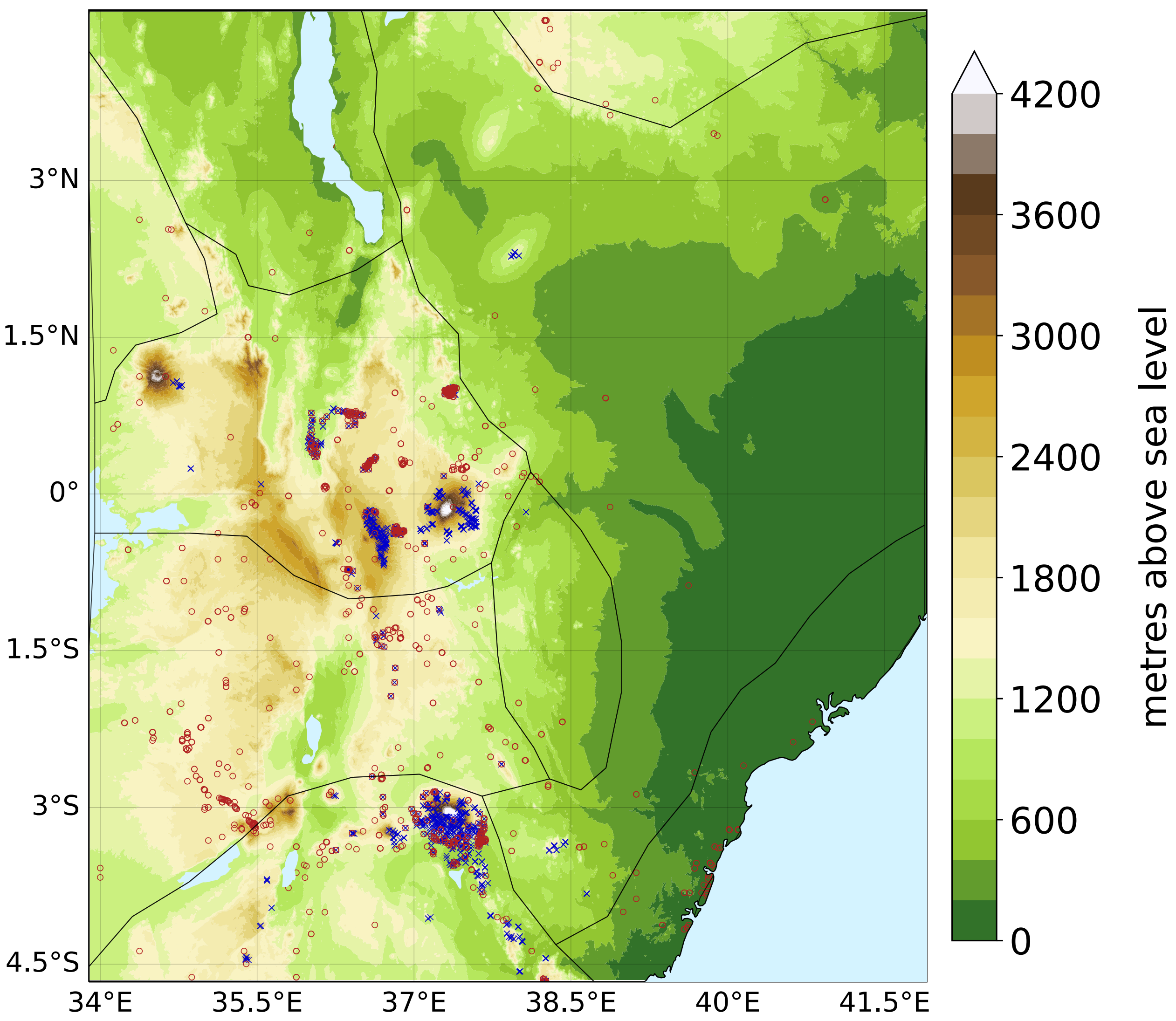

Figure S5: Presence (red circles) and absence points (blue crosses) for all investigated grass species. The shading indicates elevation in metres above sea level using the WRF topography Global Multi-resolution Terrain Elevation Data (GMTED2010) provided by USGS. Note the scarcity of data points in Eastern Kenya.

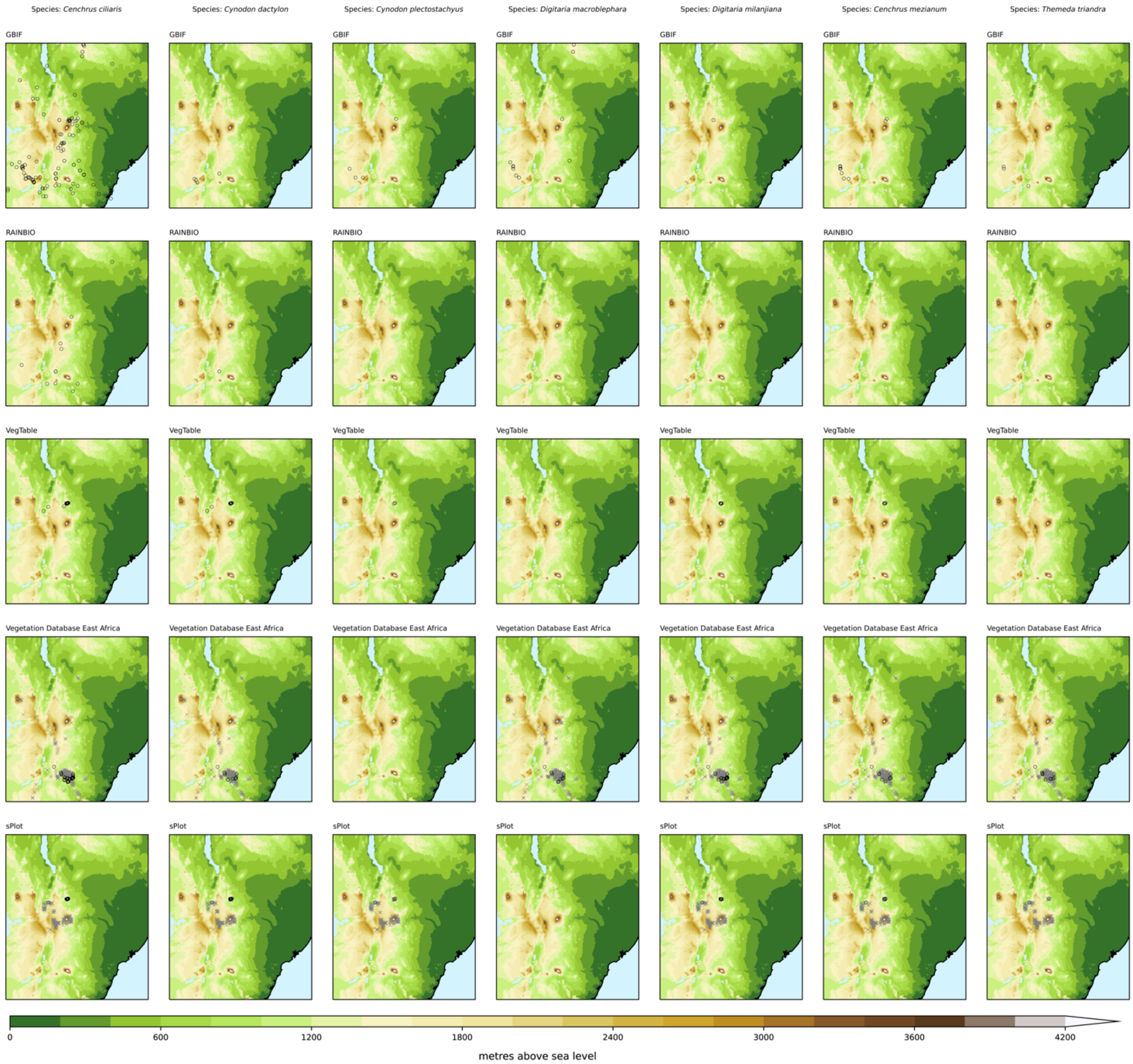

Figure S6: Presence (black circles) and absence points (grey crosses) for each species (columns) and each of the five used datasets (rows). The shading indicates elevation in metres above sea level using the WRF topography Global Multi-resolution Terrain Elevation Data (GMTED2010) provided by USGS. Empty maps indicate that no presence or absence points were recorded for the respective species and dataset.

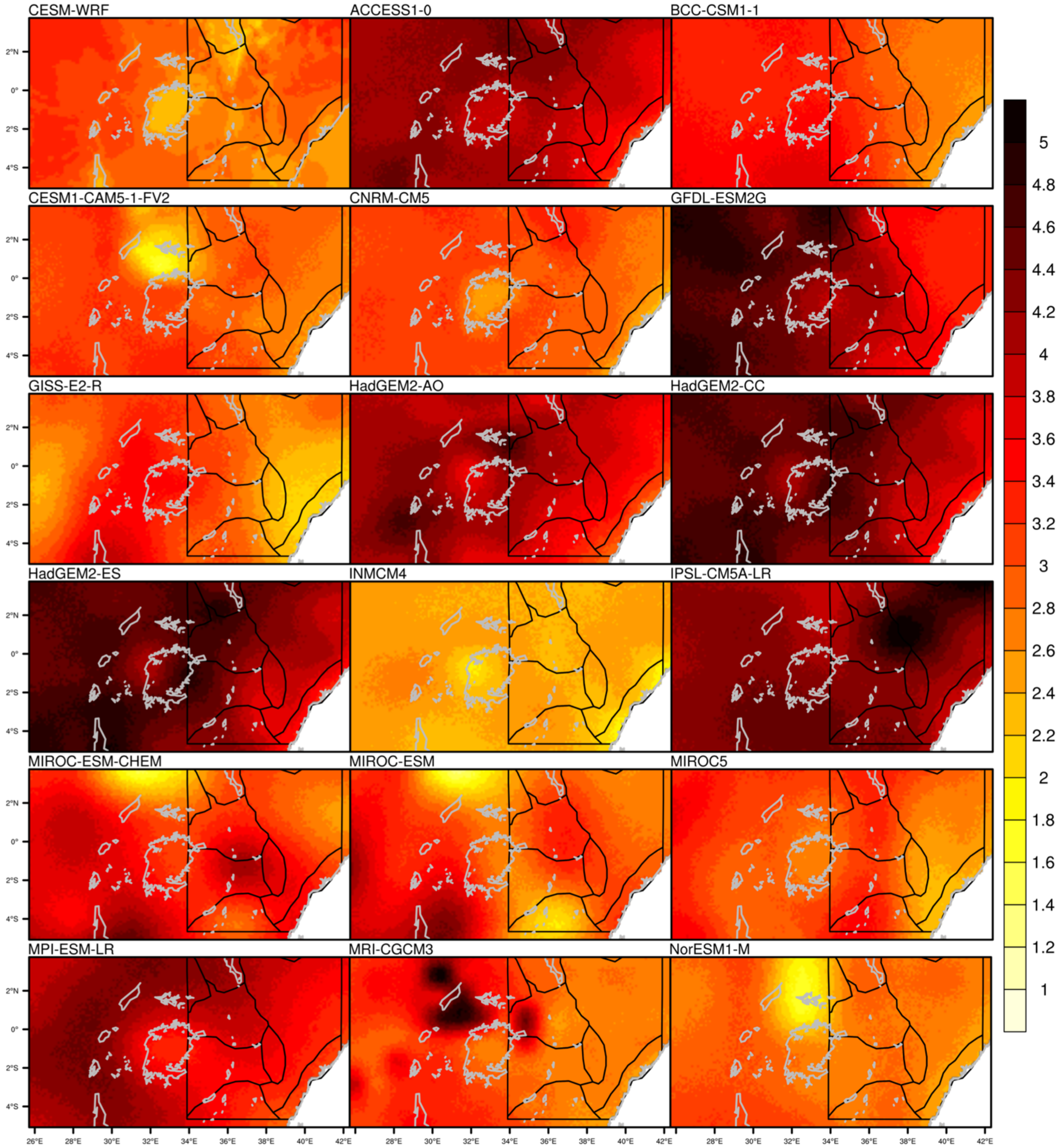

Figure S7: Difference in mean annual surface temperature in degrees Celsius between future (2071–2100) and present-day climate conditions (1981–2010) for the dynamically downscaled CESM simulation (this study, top left) and statistically downscaled CMIP5 simulations: ACCESS1-0, BCC-CSM1-1, CESM1-CAM5-FV2, CNRM-CM5, GFDL-ESM2G, GISS-E2-R, HadGEM2-AO, HadGEM2-CC, HadGEM2- ES, INMCM4, IPSL-CM5A-LR, MIROC-ESM-CHEM, MIROC-ESM, MIROC5, MPI-ESM-LR, MRI-CGCM3, and NorESM1-M (from top left to bottom right). The model acronyms are listed in Table S6.

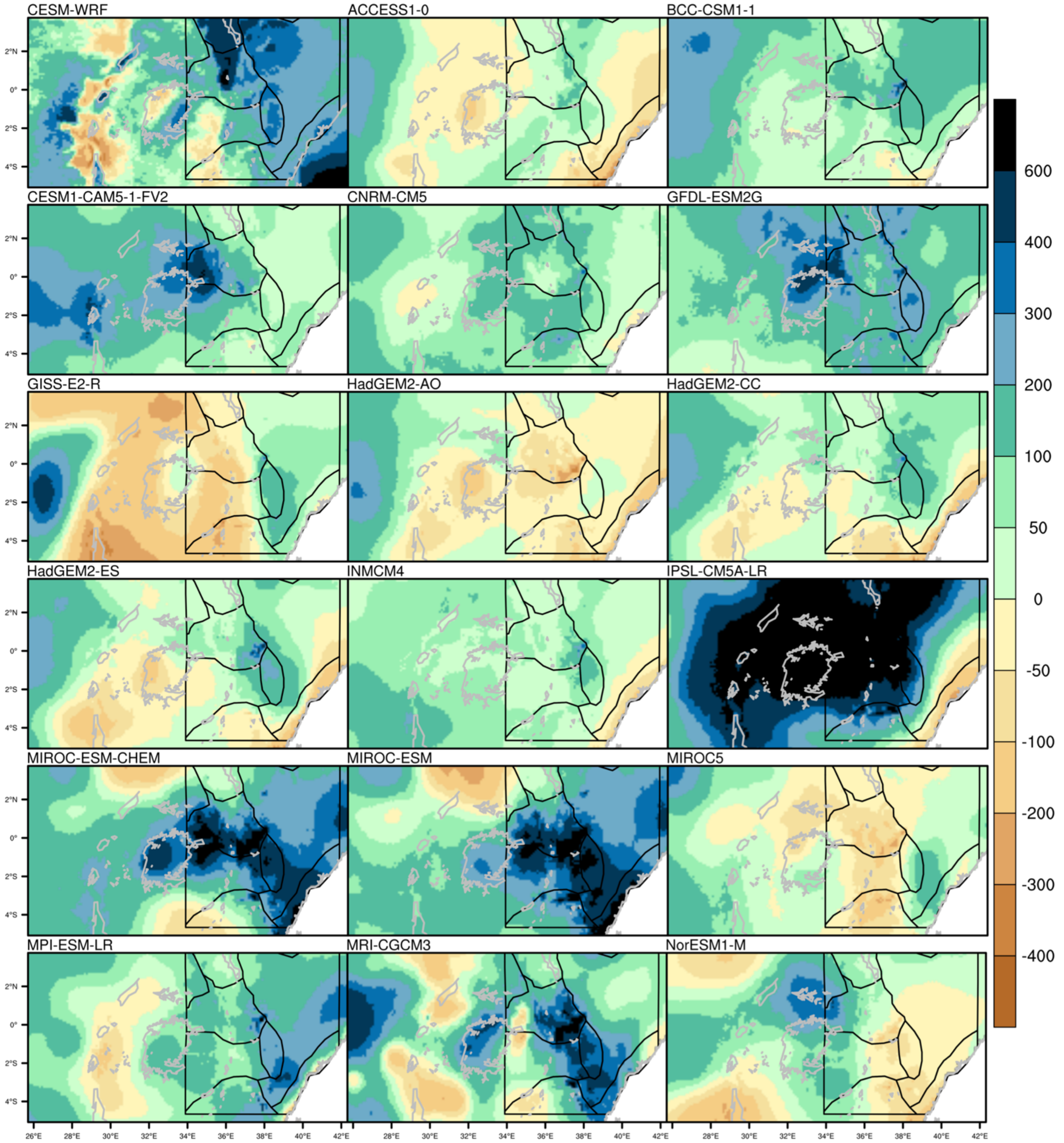

Figure S8: Difference in mean annual precipitation sum in millimetres between future (2071–2100) and present-day climate conditions (1981–2010) for the dynamically downscaled CESM simulation (this study, top left) and statistically downscaled CMIP5 simulations: ACCESS1-0, BCC-CSM1-1, CESM1-CAM5-FV2, CNRM-CM5, GFDL-ESM2G, GISS-E2-R, HadGEM2-AO, HadGEM2-CC, HadGEM2- ES, INMCM4, IPSL-CM5A-LR, MIROC-ESM-CHEM, MIROC-ESM, MIROC5, MPI-ESM-LR, MRI-CGCM3, and NorESM1-M (from top left to bottom right). The model acronyms are listed in Table S6.

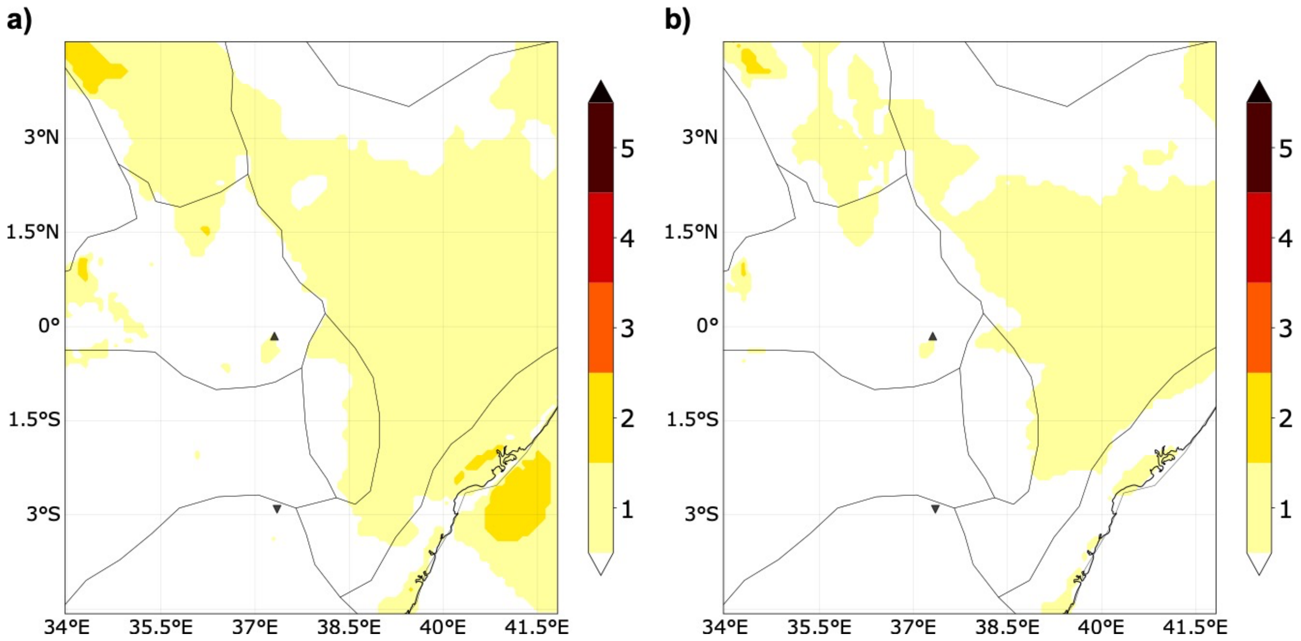

Figure S9: Novel climates for the future under RCP8.5 compared to the present for the study region. The shading indicates the number of variables (out of the five presented in Section "Changes in bioclimatic predictors under the high-emission scenario RCP8.5" and Section "Species data and species distribution models") that exceed the minimum or maximum values of the climatic range at a) only the grid points where actual presence and absence points are available and b) at all grid points in the study region. The upward-pointing black triangle indicates the location of Mount Kenya, and the downward-pointing one marks Mount Kilimanjaro.

References

1. Ndung'u, P. L. N., Wasonga, V. O., Mnene, W. N., Koech, O. K. & Yazan Elhadi, A. M. Community perception of importance, trends, and variations of indigenous grasses in Southern Kenya | RUFORUM Institutional Repository. in vol. 14 807–817 (2016).
2. Muyekho, F. N., Borrion, A. T. & Khan, Z. R. *A primer on grass identification and their uses in Kenya*. (Icipe Science Press, 2004).
3. Kirwa, E. C. Evaluation of grass ecotypes for potential use in reseeding of pastoral fields in the arid and semi-arid lands of Kenya. (University of Nairobi, 2019).
4. Tadono, T., Ishida, H., Oda, F., Naito, S., Minakawa, K. & Iwamoto, H. Precise global DEM generation by ALOS PRISM. in *ISPRS Annals of the Photogrammetry, Remote Sensing and Spatial Information Sciences* vol. II–4 71–76 (Copernicus GmbH, 2014).
5. Wildlife Conservation Society - WCS & Center for International Earth Science Information Network - CIESIN - Columbia University. Last of the wild project, version 2, 2005 (LWP-2): global human footprint dataset (IGHP). (2005).
6. Hansen, M. C., Potapov, P. V., Moore, R., Hancher, M., Turubanova, S. A., Tyukavina, A., Thau, D., Stehman, S. V., Goetz, S. J., Loveland, T. R., Kommareddy, A., Egorov, A., Chini, L., Justice, C. O. & Townshend, J. R. G. High-resolution global maps of 21st-century forest cover change. *Science* **342**, 850–853 (2013).
7. Hengl, T., Heuvelink, G. B. M., Kempen, B., Leenaars, J. G. B., Walsh, M. G., Shepherd, K. D., Sila, A., MacMillan, R. A., Jesus, J. M. de, Tamene, L. & Tondoh, J. E. Mapping soil properties of Africa at 250 m resolution: random forests significantly improve current predictions. *PLOS ONE* **10**, e0125814 (2015).
8. Poggio, L., de Sousa, L. M., Batjes, N. H., Heuvelink, G. B. M., Kempen, B., Ribeiro, E. & Rossiter, D. SoilGrids 2.0: producing soil information for the globe with quantified spatial

uncertainty. *SOIL* **7**, 217–240 (2021).

9. Masek, J. G., Vermote, E. F., Saleous, N. E., Wolfe, R., Hall, F. G., Huemmrich, K. F., Gao, F., Kutler, J. & Lim, T.-K. A Landsat surface reflectance dataset for North America, 1990-2000. *IEEE Geosci. Remote Sens. Lett.* **3**, 68–72 (2006).

10. Vermote, E., Justice, C., Claverie, M. & Franch, B. Preliminary analysis of the performance of the Landsat 8/OLI land surface reflectance product. *Remote Sens. Environ.* **185**, 46–56 (2016).
